# Prognostic nomograms and Aggtrmmns scoring system for predicting overall survival and cancer‐specific survival of patients with kidney cancer

**DOI:** 10.1002/cam4.2916

**Published:** 2020-02-22

**Authors:** Yuan Zhou, Rentao Zhang, Yinman Ding, Zhengquan Wang, Cheng Yang, Sha Tao, Chaozhao Liang

**Affiliations:** ^1^ Department of Urology Surgery The People's Hospital of Xuancheng City Xuancheng China; ^2^ Department of Urology Surgery The First Affiliated Hospital of Anhui Medical University Hefei China; ^3^ Anhui Medical University Hefei China

**Keywords:** kidney cancer, nomogram, prognostic score, regional lymph node, SEER, survival

## Abstract

**Background:**

Currently, the prognosis of kidney cancer depends mainly on the pathological grade or tumor stage. Clinicians have few effective tools that can personalize and adequately evaluate the prognosis of kidney cancer patients.

**Methods:**

A total of 70 481 kidney cancer patients were selected from the Surveillance, Epidemiology, and End Results database, among which patients diagnosed in 2005‐2011 (n = 42 890) were used to establish nomograms for overall survival (OS) and cancer‐specific survival (CSS), and those diagnosed in 2012‐2015 (n = 24 591) were used for external validation. Univariate and multivariate Cox analyses were used to determine independent prognostic factors. Concordance index (C‐index), receiver operating characteristic curve, and calibration curve were used to evaluate the predictive capacity of the nomograms. We further reduced subgroup classification and used propensity score matching to balance clinical informations, and analyzed the effect of other variables on survival. We established a new kidney cancer prognostic score system based on the effect of all available variables on survival. Cox proportional hazard model and Kaplan‐Meier curves were used for survival comparison.

**Results:**

Age, gender, marital status, surgery, grade, T stage, and M stage were included as independent risk factors in the nomograms. The favorable area under the curve (AUC) value (for OS, AUC = 0.812‐0.858; and for CSS, AUC = 0.890‐0.921), internal (for OS, C‐index = 0.776; and for CSS, C‐index = 0.856), and external (for OS, C‐index = 0.814‐0.841; and for CSS, C‐index = 0.894‐0.904) validation indicated that the proposed nomograms could accurately predict 1‐, 3‐, and 5‐year OS and CSS of kidney cancer patients. The Aggtrmmns prognostic scoring system based on age, gender, race, marital status, grade, TNM stage, and surgery of kidney cancer patients could stage patients more explicitly than the AJCC staging system.

**Conclusion:**

The nomogram and Aggtrmmns scoring system can predict OS and CSS in kidney cancer patients effectively, which may help clinicians personalize prognostic assessments and clinical decisions.

## Graphical abstract

Based on a large data set, we established prognostic nomograms and new scoring system to predict overall survival and cancer‐specific survival in kidney cancer patients, which may help clinicians personalize prognostic assessments and individualize clinical decisions.

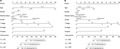

## 1 INTRODUCTION

Kidney cancer originates from the renal tubules and pelvis. Approximately 90% of kidney cancers are renal cell carcinomas, accounting for 2%‐3% of all adult malignancies.^1^ There are significant regional differences in the incidence of kidney cancer, with the highest incidence being observed in Northern America, New Zealand, Europe, and Australia and the lowest incidence being observed in Africa and the Pacific Islands.^2^ The incidence of kidney cancer is higher in males than in females, with the radio of 1.65:1, and the risk may be linked to smoking and obesity.^3^, ^4^ In 2016, an estimated 62 700 people in the United States were diagnosed with kidney cancer and 14 240 of whom died.^5^ Kidney cancer is an important disease threatening human health.

Currently, the prognosis of kidney cancer depends mainly on the pathological grade or tumor stage. The American Joint Committee on Cancer (AJCC) staging system has been widely used to predict the survival of kidney cancer patients based on the extent of tumor invasion (T), regional lymph node (N), and distant metastasis (M).^6^ Several other scoring systems, such as the Arterial Based Complexity Scoring System, nephrometry scoring systems, and Zonal NePhRO scoring system, had been proposed to standardize the evaluation of kidney cancer.^7^, ^8^, ^9^ However, these prognostic systems do not fully assess clinicopathological factors, such as age, gender, pathological grade, and marital status, which may reduce their prognostic effectiveness.

Nomogram, statistics‐based tool aggregating several independent risk factors into an intuitive graph, has been widely used in recent years to assess the prognosis for many types of cancer.^10^, ^11^, ^12^ Therefore, this study aimed to develop effective prognostic nomograms and a new scoring system based on a large data set to predict overall survival (OS) and cancer‐specific survival (CSS) of patients with kidney cancer to help clinicians provide personalized treatment recommendations.

## 2 MATERIALS AND METHODS

### 2.1 Data acquisition

Patients' data in this study were obtained from Surveillance, Epidemiology, and End Results (SEER) database (SEER 21 Regs Limited‐Field Research Data and Hurricane Katrina Impacted Louisiana Cases, released April 2019, based on the November 2018 submission, vision 8.3.5). The SEER database covers approximately 28% of cancer registries in the United States.^13^ The clinical information of cancer patients provided by SEER database greatly facilitates clinical research.

### 2.2 Study population

Patients diagnosed with kidney cancer between 2005 and 2015 from the SEER database were selected for this study. All data on kidney cancer patients were collected from hospitals, and no patient had a history of another cancer. Patients without confirmed pathological results were excluded. Available patient information, including age, gender, race, marital status, pathological grade, surgery, T (extent of tumor invasion) stage, N (regional lymph node) stage, and M (distant metastasis) stage was collected. We excluded patients in which the information mentioned above was missing.

### 2.3 Statistical analysis

We used patients diagnosed with kidney cancer between 2005 and 2011 as the primary cohort to establish nomograms. Univariate and multivariate Cox analyses were used to determine the independent prognostic factors. In the univariate Cox proportional hazard model, variables with *P* < .05 were further analyzed in the multivariate Cox proportional hazard model. Significant prognostic factors were used to establish nomograms to predict the 1‐, 3‐, and 5‐year OS and CSS rates, and the ability for survival prediction of the factors was tested by receiver operating characteristic (ROC) curve. Internal and external validations were used to evaluate the prognostic accuracy of the nomograms. We used calibration curve and concordance index (C‐index) to internal and external evaluate the predictive accuracy of the nomograms (bootstraps with 1000 resample).

Patients diagnosed between 2012 and 2015 were used as the validation cohort for external validation. Because the data sets were completed in December 2017, patients diagnosed in 2012‐2013 and in 2014‐2015 were used for the external validation of 3‐ and 1‐year survival, respectively. C‐index and area under the curve (AUC) values of 100% represent perfect predictions. Generally, the C‐index values greater than 0.7 indicate that the nomogram has a good predictive ability.^14^ In the calibration curves, the closer the prediction curve is to the observation curve, the more accurate the prognostic prediction is.

In the univariate and multivariate Cox analyses, some variables indicated no significant association with OS or CSS and thus were not included in our nomogram. To analyze the effect of these variables on survival in patients with kidney cancer, we reduced the subgroup classification to decrease distribution differences in patients diagnosed with kidney cancer between 2005 and 2015. We also used propensity score matching (PSM) to balance the clinical information and reduce statistical bias. The variables race, age, gender, marital status, grade, T stage, N stage, M stage, and surgery were matched. Cox proportional hazard models and Kaplan‐Meier curves were used for survival comparison. We further developed a new prognostic scoring system for OS and CSS in patients with kidney cancer based on the hazard ratios (HRs) of each subgroup. The new prognostic scoring system showed a total of 80 points, with 0~10 points indicating a good prognosis (stage I), 11~25 points indicating a moderate prognosis (stage II), 26~40 points indicating a poor prognosis (stage III), and 41~80 points indicating a terrible prognosis (stage IV). Kaplan‐Meier curves were used to compare the OS and CSS of patients in different stages with the new scoring system and with the AJCC staging system. All statistical analyses were performed by R software, and two‐tailed *P* < .05 were defined as statistical significance.

## 3 RESULTS

### 3.1 Baseline characteristics

A total of 70 481 patients diagnosed with kidney cancer between 2005 and 2015 were screened from the SEER database. Patients diagnosed between 2005 and 2011 (n = 42 890), as the primary cohort, were used to establish the nomograms. Patients diagnosed between 2012 and 2013 (n = 13 094) and between 2014 and 2015 (n = 14 497) were used for the external validation of 3‐ and 1‐year survival, respectively. The baseline characteristics of patients included in this study are listed in Table 1.

**Table 1:** Baseline characteristics of kidney cancer patients from SEER database (n = 70 481, 2005‐2015)

Patient characteristics	Primary cohort (n = 42 890, 2005‐2011)	Validation cohort 1 (n = 13 094, 2012‐2013)	Validation cohort 2 (n = 14 497, 2014‐2015)
	No. of patients (%)	No. of patients (%)	No. of patients (%)
Race			
White	35 624 (83.1)	10 718 (81.9)	11 810 (81.5)
Black	4721 (11.0)	1486 (11.3)	1644 (11.3)
Other	2545 (5.9)	890 (6.8)	1043 (7.2)
Age			
<50	8669 (20.2)	2544 (19.4)	2766 (19.1)
50‐59	11 705 (27.3)	3551 (27.1)	3868 (26.7)
59‐69	12 336 (28.8)	4041 (30.9)	4585 (31.6)
69‐79	7624 (17.8)	2301 (17.6)	2566 (17.7)
≥80	2556 (5.9)	657 (5.0)	712 (4.9)
Gender			
Male	27 079 (63.1)	8329 (63.6)	9225 (63.6)
Female	15 811 (36.9)	4765 (36.4)	5272 (36.4)
Marital status			
Married	28 241 (65.8)	8419 (64.3)	9255 (63.8)
Divorced	4110 (9.6)	1276 (9.7)	1421 (9.8)
Separated	471 (1.1)	180 (1.4)	182 (1.3)
Widowed	3664 (8.6)	967 (7.4)	1025 (7.1)
Single	6404 (14.9)	2252 (17.2)	2614 (18.0)
Grade			
Well	5764 (13.5)	1479 (11.3)	1548 (10.7)
Moderately	21 887 (51.0)	6708 (51.2)	7249 (50.0)
Poor	12 114 (28.2)	3864 (29.5)	4413 (30.4)
Undifferentiated	3125 (7.3)	1043 (8.0)	1287 (8.9)
T (tumor invasion)			
T1a	18 316 (42.7)	5779 (44.1)	6418 (44.3)
T1b	9972 (23.3)	2985 (22.8)	3321 (22.9)
T2	5292 (12.3)	1480 (11.3)	1508 (10.4)
T3a	4461 (10.4)	1387 (10.6)	1491 (10.3)
T3b	3991 (9.3)	1253 (9.6)	1510 (10.4)
T3c	168 (0.4)	46 (0.4)	62 (0.4)
T4	690 (1.6)	164 (1.2)	187 (1.3)
N (regional lymph node)			
No	41 106 (95.8)	12 501 (95.5)	13 915 (96.0)
Yes	1784 (4.2)	593 (4.5)	582 (4.0)
M (metastasis)			
No	39 543 (92.2)	12 096 (92.4)	13 393 (92.4)
Yes	3347 (7.8)	998 (7.6)	1104 (7.6)
Surgery			
No	930 (2.2)	365 (2.8)	448 (3.1)
Yes	41 960 (97.8)	12 769 (97.2)	14 049 (96.9)
Pathology			
Clear cell adenocarcinoma	26 012 (60.7)	8701 (66.5)	10 010 (69.0)
Adenocarcinoma with mixed subtypes	1042 (2.4)	340 (2.6)	419 (2.9)
Papillary adenocarcinoma	4745 (11.1)	1577 (12.0)	1738 (12.0)
Chromophobe cell carcinoma	1949 (4.5)	593 (4.5)	563 (3.9)
Other	1808 (4.2)	419 (3.2)	405 (2.8)
Unknown	7334 (17.1)	1464 (11.2)	1362 (9.4)

Note

The primary cohort was used to establish nomograms. The validation cohort 1 and validation cohort 2 were used for the external validation of 3‐ and 1‐y survival of nomograms, respectively.

### 3.2 Independent prognostic factors

The results of the univariate and multivariate Cox analyses of OS and CSS of the primary cohort are listed in Tables 2 and 3, respectively. According to the univariate cox analysis, the variables age, gender, marital status, grade, T stage, M stage, and surgery were significantly associated with OS and CSS. These significant variables were further entered into the multivariate Cox analysis, all of which were considered independent prognostic factors for OS and CSS (Tables 2 and 3). In the multivariate Cox analyses, with regard to OS, older age (compared with <60 years old, ≥80 years old: HR = 4.82; 95% CI = 4.47‐5.19; *P* < .001) and M1 classification (compared with M0: HR = 4.34; 95% CI = 4.12‐4.56; *P* < .001) were associated with the highest risk of death (Table 2). With regard to CSS, higher T stage (compared with T1a, T1b‐T4: HR = 2.30‐9.62; *P* < .001 for all) and M1 classification (compared with M0: HR = 5.44; 95% CI = 5.14‐5.76; *P* < .001) were related to the highest risk of death (Table 3).

**Table 2:** Univariate and multivariate Cox analyses for overall survival in the primary cohort (n = 42 890, 2005‐2011)

Patient characteristics	Univariate analysis *P* value	Multivariate analysis	
		HR (95% CI)	*P* value
Age	<.001		<.001
<50		Reference	
50‐59		1.40 (1.31‐1.49)	<.001
59‐69		1.95 (1.84‐2.08)	<.001
69‐79		3.02 (2.83‐3.21)	<.001
≥80		4.82 (4.47‐5.19)	<.001
Gender	<.001		<.001
Male		Reference	
Female		0.80 (0.77‐0.84)	<.001
Marital status	<.001		<.001
Married		Reference	
Divorced		1.37 (1.29‐1.45)	<.001
Separated		1.55 (1.32‐1.81)	<.001
Widowed		1.43 (1.35‐1.51)	<.001
Single		1.37 (1.31‐1.45)	<.001
Grade	<.001		<.001
Well		Reference	
Moderately		1.02 (0.96‐1.08)	.545
Poor		1.35 (1.27‐1.44)	<.001
Undifferentiated		2.34 (2.17‐2.52)	<.001
T stage	<.001		<.001
T1a		Reference	
T1b		1.41 (1.35‐1.49)	<.001
T2		1.73 (1.63‐1.83)	<.001
T3a		2.21 (2.09‐2.34)	<.001
T3b		2.57 (2.42‐2.72)	<.001
T3c		3.76 (3.15‐4.94)	<.001
T4		3.99 (3.63‐4.39)	<.001
M stage	<.001		<.001
M0		Reference	
M1		4.34 (4.12‐4.56)	<.001
Surgery	<.001		<.001
Yes		Reference	
No		3.21 (2.97‐3.48)	<.001
Race	.321		
N stage	.887		
Pathology	.842		

Abbreviations: CI, confidence interval; HR, hazard ratio.

**Table 3:** Univariate and multivariate Cox analyses for cancer‐specific survival in the primary cohort (n = 42 890, 2005‐2011)

Patient characteristics	Univariate analysis *P* value	Multivariate analysis	
		HR (95% CI)	*P* value
Age	<.001		<.001
<50		Reference	
50‐59		1.17 (1.08‐1.26)	<.001
59‐69		1.44 (1.34‐1.55)	<.001
69‐79		1.88 (1.73‐2.04)	<.001
≥80		2.49 (2.25‐2.76)	<.001
Gender	.001		<.001
Male		Reference	
Female		0.90 (0.86‐0.95)	.001
Marital status	<.001		<.001
Married		Reference	
Divorced		1.19 (1.10‐1.28)	<.001
Separated		1.42 (1.16‐1.75)	<.001
Widowed		1.22 (1.13‐1.33)	<.001
Single		1.19 (1.11‐1.27)	<.001
Grade	<.001		<.001
Well		Reference	
Moderately		1.10(0.99‐1.22)	.078
Poor		1.97 (1.77‐2.18)	<.001
Undifferentiated		3.51 (3.14‐3.92)	<.001
T stage	<.001		<.001
T1a		Reference	
T1		2.30 (2.11‐2.52)	<.001
T2		3.93 (3.60‐4.29)	<.001
T3a		5.22 (4.78‐5.70)	<.001
T3b		6.28 (5.75‐6.85)	<.001
T3c		9.10 (8.08‐10.24)	<.001
T4		9.62 (7.90‐11.72)	<.001
M stage	<.001		<.001
M0		Reference	
M1		5.44 (5.14‐5.76)	<.001
Surgery	<.001		<.001
Yes		Reference	
No		3.83 (3.49‐4.20)	<.001
Race	.120		
N stage	.801		
Pathology	.350		

Abbreviations: CI, confidence interval; HR, hazard ratio.

### 3.3 Establishment and validation of the prognostic nomograms

**Figure 1:**
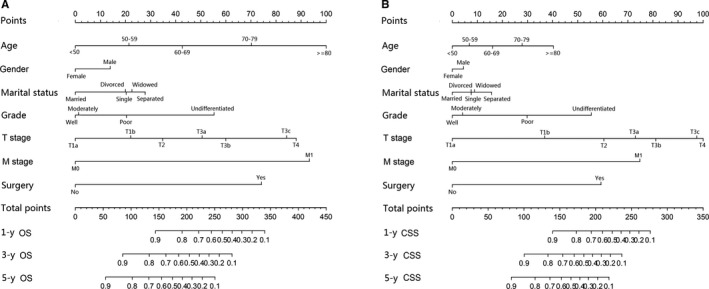
Nomograms to predict the 1‐, 3‐, and 5‐y overall survival (OS) and cancer‐specific survival (CSS) rates of kidney cancer patients. A, 1‐, 3‐, and 5‐y OS rate; (B) 1‐, 3‐, and 5‐y CSS rate

**Figure 2:**
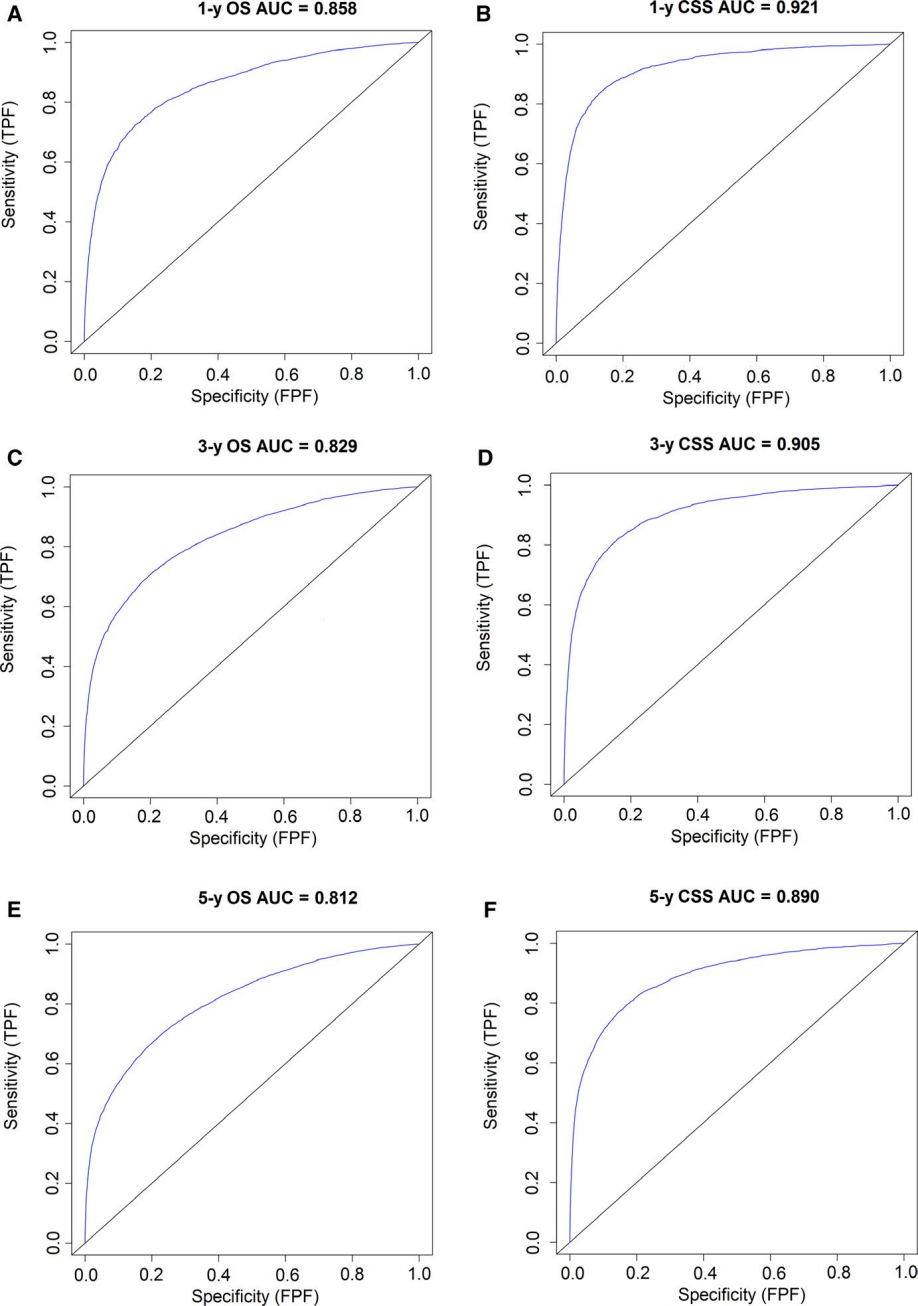
Receiver operating characteristic curve to test the predictive ability for 1‐, 3‐, and 5‐y overall survival (OS) and cancer‐specific survival (CSS) using variables from our developed nomograms in kidney cancer patients. Receiver operating characteristic curve for predicting (A) 1‐y OS; (B) 1‐y CSS; (C) 3‐y OS; (D) 3‐y CSS; (E) 5‐y OS; (F) 5‐y CSS

**Figure 3:**
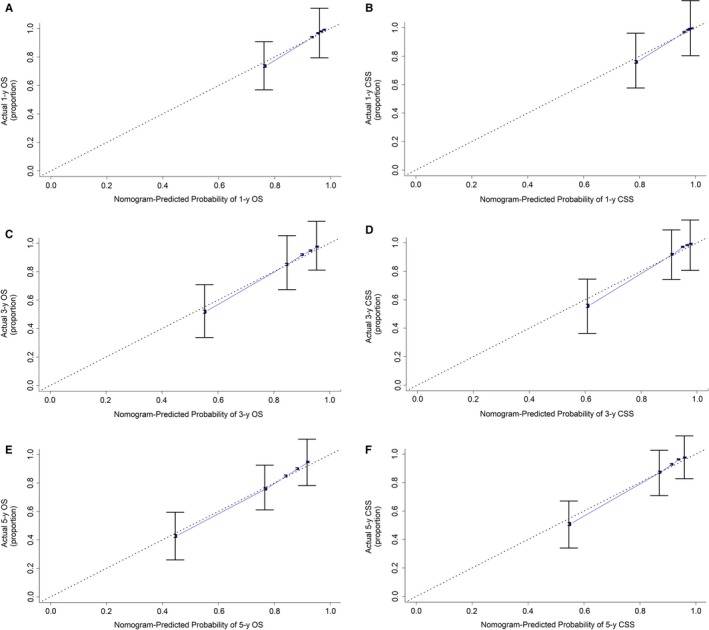
The calibration curves to internal verify the nomograms in the primary data set. Calibration curves for predicting (A) 1‐y OS; (B) 1‐y CSS; (C) 3‐y OS; (D) 3‐y CSS; (E) 5‐y OS; (F) 5‐y CSS

The independent prognostic factors were used to establish nomograms to predict the 1‐, 3‐, and 5‐year OS and CSS rates of kidney cancer patients (Figure 1). The predictive ability for 1‐, 3‐, and 5‐year OS and CSS of kidney cancer patients using variables from our developed nomograms was tested by ROC curves. The favorable 1‐, 3‐, and 5‐year AUC values (for OS, AUC = 0.858, 0.829, and 0.812, respectively; and for CSS, AUC = 0.921, 0.905, and 0.890, respectively) indicated good ability for survival prediction of variables in kidney cancer patients (Figure 2). The accuracy of nomograms was evaluated internally with C‐index values and correction curve. The favorable C‐index values (for OS, C‐index = 0.776, 95% CI = 0.772‐0.780; and for CSS, C‐index = 0.856, 95% CI = 0.852‐0.860) indicated that the proposed nomograms could accurately predict the 1‐, 3‐, and 5‐year OS and CSS of kidney cancer patients. On the other hand, the 1‐, 3‐, and 5‐year calibration curves of OS and CSS demonstrated good consistency between predicted survival and observed survival (Figure 3), which could also prove the validity of prognostic nomograms.

**Figure 4:**
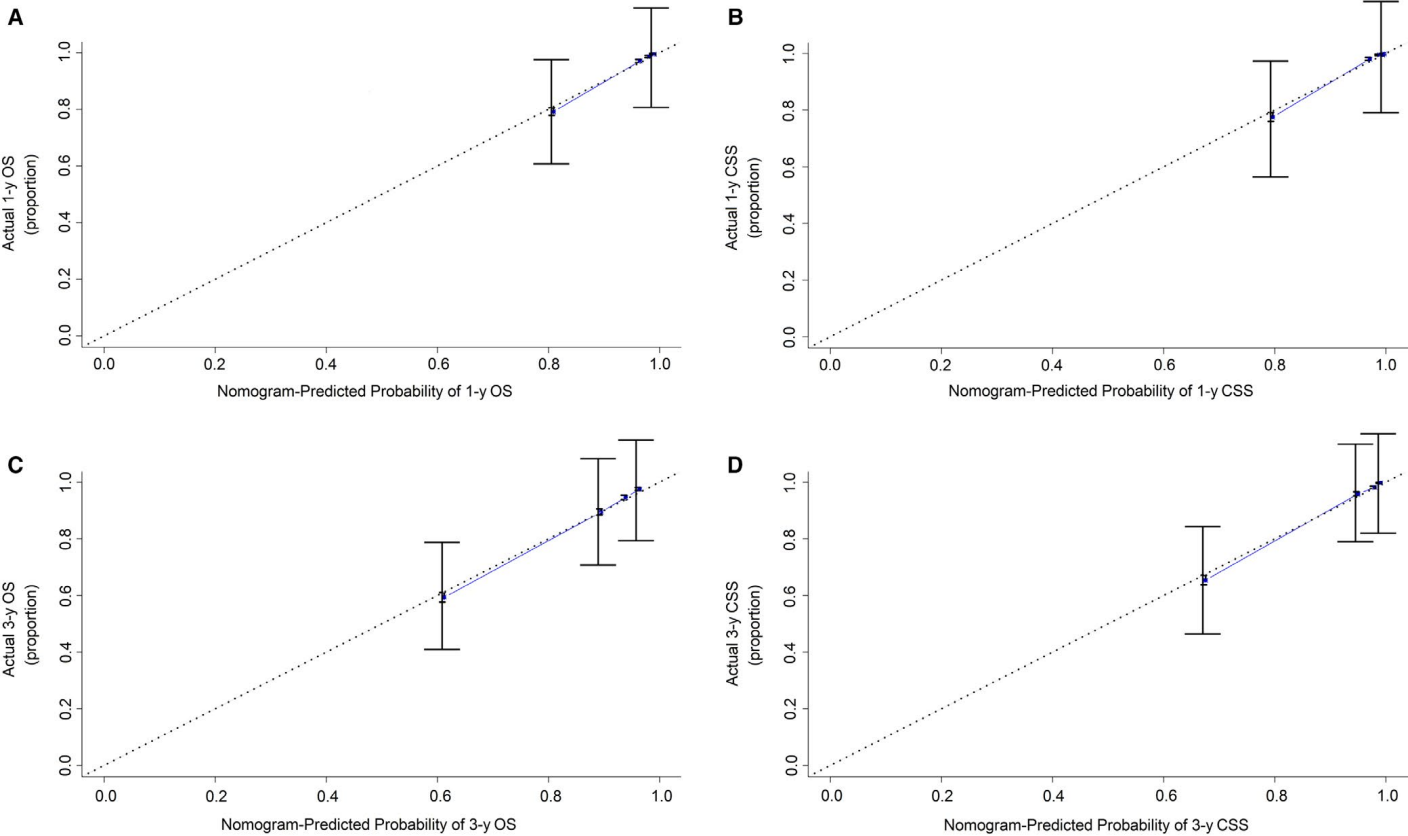
The calibration curves to external verify the nomograms in the validation data set. Calibration curves for predicting (A) 1‐y OS; (B) 1‐y CSS; (C) 3‐y OS; (D) 3‐y CSS

We further used C‐index and calibration curve for the external verification of the nomograms. The excellent C‐index values (OS: 1‐year value = 0.841, 95% CI = 0.829‐0.853; 3‐year value = 0.814, 95% CI = 0.804‐0.824; CSS: 1‐year value = 0.904, 95% CI = 0.894‐0.914; 3‐year value = 0.894, 95% CI = 0.886‐0.902) and calibration curves indicated good agreements between prediction and actual observation (Figure 4). These internal and external validations indicate that the prognostic nomograms we proposed are effective and accurate in evaluating the 1‐, 3‐, and 5‐year OS and CSS of patients with kidney cancer.

### 3.4 The effect of other variables on survival

**Figure 5:**
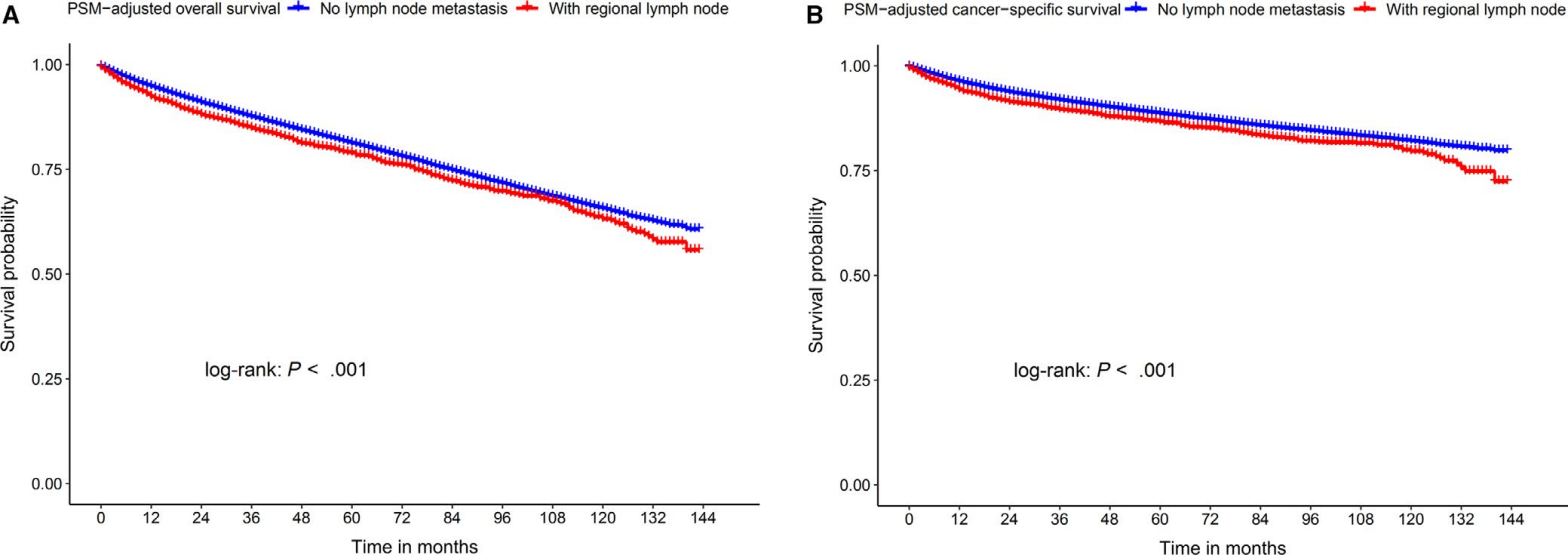
The comparison of overall survival and cancer‐specific survival between kidney cancer patients with and without regional lymph node metastasis. Kaplan‐Meier curves for comparison (A) overall survival; (B) cancer‐specific survival. PSM, propensity score matching

We used univariate and multivariate Cox analyses to determine the independent prognostic factors, the variables N stage, race and pathology had no statistical significance in OS and CSS (*P* > .05). To analyze the effects of the abovementioned variables on survival in kidney cancer patients, we reduced the subgroup classification to decrease distribution differences in patients diagnosed with kidney cancer between 2005 and 2015, and the baseline characteristics of the patients are listed in Table 4. We further used PSM to balance the clinical information and reduce the statistical bias and found kidney cancer patients with regional lymph node metastasis had a slightly increased risk of death compared to those without regional lymph node metastasis (overall death: HR = 1.09; 95% CI = 1.00‐1.17; *P* = .034; cancer‐specific death: HR = 1.13; 95% CI = 1.02‐1.25; *P* = .018), and black patients had the worst prognosis of all the races (Table 5). We generated Kaplan‐Meier curves to compare the OS and CSS of kidney cancer patients with and without regional lymph node metastasis (Figure 5). Regarding the pathological types, the *P* values were not significant, even after reducing differences between subgroups and matching clinical information (Table 5).

**Table 4:** Baseline characteristics of kidney patients after reduced subgroup classification (n = 70 481, 2005‐2015)

Patient characteristics	No. of patients (%)
Race	
White	58 152 (82.5)
Black	7851 (11.1)
Other	4478 (6.4)
Gender	
Male	44 633 (63.3)
Female	25 848 (36.7)
Age	
<60	33 103 (47.0)
60‐80	20 962 (29.7)
>80	16 416 (23.3)
Marital status	
Married	45 915 (65.1)
Sep/Div/Wid	13 296 (18.9)
Single	11 270 (16.0)
Surgery	
Yes	68 738 (97.5)
No	1743 (2.5)
Grade	
Well	8791 (12.5)
Moderately	35 844 (50.9)
Poor	20 391 (28.9)
Undifferentiated	5455 (7.7)
T (tumor invasion)	
T1	46 791 (66.4)
T2	8280 (11.7)
T3	14 369 (20.4)
T4	1041 (1.5)
N (regional lymph node)	
Yes	2959 (4.2)
No	67 522 (95.8)
M (metastasis)	
Yes	5449 (7.7)
No	65 032 (92.3)

**Table 5:** The comparison of overall death risk and all cancer‐specific death risk on survival of kidney cancer patients for variables not included in the nomograms

Patient characteristics	Overall HR (95% CI)	*P* value	Cancer‐specific HR (95% CI)	*P* value
N (regional lymph node)				
Yes vs None	1.09 (1.00‐1.17)	.034	1.13 (1.02‐1.25)	.018
Race				
Black vs White	1.28 (1.22‐1.35)	<.001	1.27 (1.18‐1.36)	<.001
Other vs White	0.90 (0.85‐0.96)	.002	0.96 (0.89‐1.05)	.372
Pathology				
B vs A	1.08 (0.98‐1.20)	.127	1.13 (0.99‐1.30)	.060
C vs A	1.01 (0.96‐1.06)	.700	1.04 (0.97‐1.11)	.227
D vs A	1.01 (0.93‐1.09)	.819	1.06 (0.96‐1.18)	.252
E vs A	1.07 (0.98‐1.16)	.128	1.16 (1.03‐1.30)	.120
F vs A	1.00 (0.96‐1.05)	.988	1.02 (0.96‐1.08)	.534

Note

The comparisons between groups were performed after propensity matching score adjusted.

Abbreviations: A, clear cell adenocarcinoma; B, adenocarcinoma with mixed subtypes; C, papillary adenocarcinoma; CI, confidence interval; D, chromophobe cell carcinoma; E, other types; F, unknown; HR, hazard ratio.

### 3.5 Establishment of Aggtrmmns prognostic scoring system

**Figure 6:**
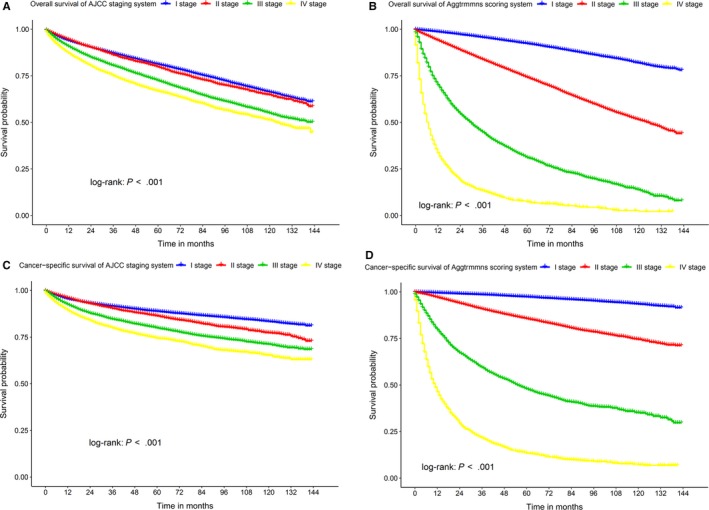
The comparison of overall survival (OS) and cancer‐specific survival (CSS) based on the stages of Aggtrmmns scoring system and AJCC staging system. Kaplan‐Meier curves for comparison OS in (A) AJCC and (B) Aggtrmmns staging system and CSS in (C) AJCC and (D) Aggtrmmns staging system.

According to the HRs of the subgroups shown in Tables 2, 3, and 5, we established a new score system to evaluate OS and CSS of patients with kidney cancer. The prognostic score system was based on age, gender, race, marital status, grade, TNM stage, and surgery of kidney cancer patients. The scores of each subgroup are listed in Table 6. Based on the first letter of each of the above variables, we named this system the Aggtrmmns scoring system. We used Kaplan‐Meier curves to compare the OS and CSS of kidney cancer patients determined with the new scoring system to those determined with the AJCC staging system (Figure 6) and found that OS rates and CSS rates of kidney cancer patients with different stages were more explicit when our scoring system was used (Table 7).

**Table 6:** The risk scores of Aggtrmmns prognostic scoring system for overall death and specific death of patients with kidney cancer

Patient characteristics	All‐cause death points	Cancer‐specific death points
Age		
<50	0	0
50‐59	2	1
59‐69	5	2
69‐79	11	3
≥80	20	6
Gender		
Male	1	0
Female	0	0
Marital status		
Married	0	0
Divorced	2	1
Separated	3	2
Widowed	2	1
Single	2	1
Grade		
Well	0	0
Moderately	0	0
Poor	2	4
Undifferentiated	7	10
Race		
White	1	0
Black	2	1
Other	0	0
T stage		
T1a	0	0
T1b	2	5
T2	4	11
T3a	6	16
T3b	8	20
T3c	15	31
T4	16	33
N stage		
No	0	0
Yes	1	0
M stage		
M0	0	0
M1	18	17
Surgery		
No	12	11
Yes	0	0

Note

The Aggtrmmns prognostic scoring system with a total of 80 points, with 0~10 points indicating a good prognosis (stage I), 11~25 points indicating a moderate prognosis (stage II), 26~40 points indicating a poor prognosis (stage III), and 40~80 points indicating the tumor was deadly to survival (stage IV).

**Table 7:** The comparison of overall and cancer‐specific survival rates of kidney cancer patients between AJCC staging system and Aggtrmmns scoring system

Stage	Survival time	AJCC staging system			Aggtrmmns scoring system			
		OS		CSS	OS		CSS	
		No.	Rate (%)	Rate (%)	No.	Rate (%)	No.	Rate (%)
Stage I	1‐y	44 046	93.9	95.5	37 125	98.6	40 529	99.5
	3‐y		87.1	91.6		95.6		98.5
	5‐y		81.1	88.8		92.3		97.3
Stage II	1‐y	7050	94.4	95.9	25 410	94.0	20 253	97.1
	3‐y		86.1	90.4		83.8		90.8
	5‐y		79.8	86.4		74.1		85.6
Stage III	1‐y	13 542	90.7	92.5	6098	70.0	6234	79.8
	3‐y		80.4	84.6		44.7		59.4
	5‐y		72.4	79.9		31.0		47.9
Stage IV	6‐mo	5843	91.7	93.2	1848	50.1	3465	63.7
	1‐y		87.1	89.4		33.5		46.3
	3‐y		75.0	79.9		13.6		21.6

## 4 DISCUSSION

Nomogram has become increasingly popular for its important role in personalized cancer prediction,^15^ which can help clinicians predict the survival of cancer patients with individual information. At present, the AJCC staging system and Fuhrman pathological grading system are widely used to evaluate the prognosis of kidney cancer patients, but the clinical information of patients cannot be individually and fully evaluated, which may limit the prediction effect. Therefore, this study aimed to establish prognostic nomograms to accurately and effectively predict the OS and CSS of patients with kidney cancer. As a result, nomograms for 1‐, 3‐, and 5‐year OS and CSS were established, and their accuracy was demonstrated by internal and external validations. The prognostic nomograms we proposed might promote the popularization of individualized treatment and survival assessment for kidney cancer patients.

The variables age, gender, marital status, grade, T stage, M stage, and surgery were included in our nomogram as independent prognostic factors. As shown in our nomogram, older patients with kidney cancer had worse OS and CSS, and the risk of death was more significant in CSS. In general, older patients have more chronic diseases and less resistance to disease, which may lead to a higher risk of overall death and cancer‐specific death. The effect of gender on kidney cancer patient survival was previously reported, as a group, kidney cancer in males presented with larger, higher stage, higher grade and faster progression than in females,^16^, ^17^ and females had a better chance of recovering renal function than males after radical nephrectomy.^18^ Marital status has been proven to be an independent prognostic factor and affects the survival of many types of cancer patients, such as prostate cancer, lung cancer, breast cancer, non‐Hodgkin lymphoma, and ovarian cancer.^19^ In these types of cancer, married patients have better OS and CSS than those with other marital conditions.^19^ A previous study on kidney cancer showed that married patients had the highest survival rate, followed by those who were divorced/ separated, and the widowed patients had the lowest survival rate.^20^ However, the above study did not analyze separated and divorced patients separately, nor did they use PSM to balance the clinical variables. In our study, separated patients with kidney cancer had the highest risk of death (compared with married, OS: HR = 1.55; 95% CI = 1.32‐1.81; CSS: HR = 1.42; 95% CI = 1.16‐1.75; *P* < .001 for all). We further used PSM to balance the clinical variables and analyzed the impact of different marital status on the survival of kidney cancer patients. The variables race, age, gender, marital status, grade, T stage, N stage, M stage, pathology, and surgery were matched. We found separated status had the most adverse impact on OS (referent: married, HR: 1.73, 95% CI = 1.49‐2.01; *P* < .001) and CSS (referent: married, HR: 1.71, 95% CI = 1.40‐2.01; *P* < .001), and married patients had the best OS and CSS (Table S1). The effects of gender and marital status on the OS and CSS of kidney cancer patients in our study were consistent with those from previous reports, which could confirm the authenticity of our nomogram on the other hand.

The variables N stage, race, and pathology had no statistical significance in the univariate Cox analysis and thus were not included in the nomograms. After reducing the subgroup classification to decrease distribution differences and using PSM to reduce statistical bias, we found kidney cancer patients with regional lymph node metastasis had a slightly increased risk of death compared to those without regional lymph node metastasis (HR = 1.09 for OS and HR = 1.13 for CSS; *P* < .05 for all). Whether or not implement regional lymph node dissection after radical nephrectomy has been controversial. A prospective study by European Organization in 2008 reported that regional lymph node dissection after radical nephrectomy had no significant effect on survival or cancer recurrence.^21^ However, many thought that most patients in the trial had low‐stage tumors with a negligible risk of lymph node involvement.^22^ Recent views stated that lymph node dissection was not recommended for localized kidney cancer, and patients with stage T3‐T4, a high Fuhrman grade, sarcomatoid features, and/or coagulation tumor necrosis might benefit from regional lymph node dissection.^23^ However, due to the absence of information on surgical methods in the SEER database, we could not perform further statistical analysis. Psychotherapy is an important aspect of cancer treatment, and stress might accelerate the cancer process.^24^ Regional lymph node metastasis in many types of cancer has a significant impact on survival, which might make patients anxious and negatively affect their survival. Our study revealed that regional lymph node metastasis had a slight effect on survival in patients with kidney cancer, which could reduce panic of patients and improved the effect of treatment. In the analysis of kidney cancer patients for race, black patients had the worst survival, followed by white patients, and the survival of other race was the best. Our results are consistent with those from a previous study, showing that black patients with kidney cancer have a high incidence and a low survival rate, while the trend was reversed for Asians/Pacific Islanders.^25^

The Aggtrmmns prognostic scoring system we proposed combined the clinicopathological information of kidney cancer patients with a total of 80 points, among which the TNM stage accounts for 35 points in OS and 50 points in CSS. Therefore, the Aggtrmmns prognostic scoring system could assess the OS and CSS of kidney cancer patients more efficiently than the AJCC staging system. Combined with the prognostic nomograms and Aggtrmmns scoring system, we could evaluate the OS and CSS of patients with kidney cancer more effectively and accurately, which might be helpful for clinicians in formulating treatment plans and adjusting follow‐up strategies. For example, in kidney cancer patients with poor prognosis, we could implement additional treatments and reduce follow‐up time to monitor the disease more effectively.

Our study still had some limitations. First, the treatment method used, such as radiation, chemotherapy, and immunotherapy, might have affected the survival of patients with kidney cancer, and the absence of treatment information in the SEER database might have introduced bias to our results. Second, renal function is an important factor affecting the survival of kidney cancer patients. If information on renal function or hemodialysis was added, our prognostic nomograms and scoring system would be more complete. Last, because our study was essentially retrospective and decreased statistical power in the subgroup analysis, larger and prospective studies are needed to prove our results.

## 5 CONCLUSIONS

With data from a large set, we developed prognostic nomograms and Aggtrmmns scoring system and demonstrated that those tools could make individual OS and CSS predictions for patients with kidney cancer. The proposed survival models of kidney cancer may help clinicians make individual therapies and adjust follow‐up strategies. Kidney cancer patients with regional lymph node metastasis have a slight but significant increase in the risk of overall death and cancer‐specific death compared to those without regional lymph node metastasis, therefore regional lymph node metastasis may be an indicator for radical nephrectomy surgery. Because of the limitation of the present study, larger and prospective studies are needed to validate our findings.

## CONFLICT OF INTEREST

The authors have no conflict of interest.

## AUTHOR CONTRIBUTION

Conceptualization: Chaozhao Liang; data curation: Yuan Zhou, Rentao Zhang; formal analysis: Yuan Zhou, Zhengquan Wang, Yinman Ding; funding acquisition: Chaozhao Liang; methodology: Yuan Zhou, Rentao Zhang; supervision: Chaozhao Liang; validation: Rentao Zhang, Sha Tao; writing—original draft: Yuan Zhou, Rentao Zhang; writing: review and editing: Yuan Zhou, Cheng yang.

## Data Availability

All data examined in this study were obtained from the SEER database with the purpose of research. This study did not include any human or animal participants.
