# Supplementary material for: Prognostic nomograms and Aggtrmmns scoring system for predicting overall survival and cancer‐specific survival of patients with kidney cancer
Source: Cancer Med. 2020 Feb 22;9(8):2710–22. doi: 10.1002/cam4.2916 (PMC7163106; doi:10.1002/cam4.2916)
Supplement: Supplementary file 1 [file CAM4-9-2710-s001.docx]

**TableS1:** The comparison of overall death risk and all cancer-specific death risk for marital status (n=70,481, 2005-2015).

| **Patient characteristics** | **All-cause**  **HR (95% CI)** | ***P* value** | **Cancer-specific**  **HR (95% CI)** | ***P* value** |
| --- | --- | --- | --- | --- |
| Marital Status |  |  |  |  |
| Married | Reference |  | Reference |  |
| Divorced | 1.41(1.33-1.49) | <0.001 | 1.25(1.16-1.34) | <0.001 |
| Separated | 1.73(1.49-2.01) | <0.001 | 1.71(1.40-2.01) | <0.001 |
| Widowed | 1.42(1.34-1.50) | <0.001 | 1.28(1.17-1.39) | <0.001 |
| Single | 1.41(1.35-1.48) | <0.001 | 1.20(1.12-1.28) | <0.001 |

**Note:** The distribution of subgroups were based on Table 1, and all the comparisons were performed after propensity matching score adjusted. Abbreviations:HR=hazard ratio, CI=confidence interval.
